# Prevalence of Metabolic Syndrome and its Associated Factors among Multi-ethnic Adults in Rural Areas in Xinjiang, China

**DOI:** 10.1038/s41598-017-17870-5

**Published:** 2017-12-15

**Authors:** Heng Guo, Xiang Gao, Rulin Ma, Jiaming Liu, Yusong Ding, Mei Zhang, Jingyu Zhang, Lati Mu, Jia He, Yizhong Yan, Jiaolong Ma, Shuxia Guo, Sheng Wei

**Affiliations:** 1 Department of Epidemiology and Biostatistics, School of Public Health, Tongji Medical College, Huazhong University of Science and Technology, Wuhan, Hubei 430030 China; 2 Department of Public Health, Shihezi University School of Medicine, Shihezi, Xinjiang 832000 China; 3 Department of Nutritional Sciences, The Pennsylvania State University 109 Chandlee Lab, University Park, PA 16801 USA

## Abstract

Metabolic syndrome (MetS) has become a global public health problem affecting all nations and races. Few studies on the epidemic of metabolic syndrome (MetS) examined multi-ethnic adults in rural areas in Xinjiang, China. We thus investigated the prevalence and risk factors of MetS there. A cross-sectional study was performed in a representative sample of 15020 rural multi-ethnic adults from 2009 to 2010. Four widely used criteria (ATPIII\IDF\JIS\CDS) were used to measure the prevalence of MetS. Multiple logistic regression analysis was used to explore the risk factors of MetS. The age-adjusted prevalence of MetS was 14.43%, 21.33%, 26.50%, and 19.89% based on the ATP III, IDF, JIS and CDS criterion, respectively. The prevalence of MetS was higher in women and increased with age. According to JIS criterion, the prevalence of components in MetS was 57.75% for abdominal obesity, 44.05% for elevated blood pressure, 40.98% for reduced HDL-cholesterol, 23.33% for elevated triglycerides, 18.95% for raised fasting plasma glucose. Lower consumption of vegetables, milk, and higher consumption of red meat were associated with higher likelihood of having MetS. The prevalence of MetS in Xinjiang rural multi-ethnic adults was high. Diet factors were associated with the prevalence of MetS.

## Introduction

Metabolic syndrome (MetS) is a reflection of the body’s metabolic disorders that cause chronic damage to organs^1,2^. It directly increases the risk of cardiovascular disease^1^, type 2 diabetes mellitus and all-cause mortality^3^. The prevalence of MetS is high and on the rise in both developing and developed countries. The International Diabetes Federation (IDF) estimates that one-quarter of the world’s adult population may have MetS^4^. In China, the prevalence of MetS has increased 37% from 2001 to 2009 (13.3% in 1993 vs 18.2% in 2009) according to NCEP- ATPIII criterion^5,6^, translating to approximately 200 million adults with MetS. As the high-calorie dietary pattern and sedentary lifestyle have become increasingly popular, this figure will continue to grow in China.

Previous studies found that dietary factors, smoking and drinking were associated with MetS, but the conclusions were inconsistent in different regions and populations^7–10^. The Xinjiang Uyghur autonomous region is a multi-ethnic area, which is located in the northwest of China approximately 2,000 miles from Beijing. The Uyghur, Han, Kazakh, and Kyrgyz were main ethnic groups, these four ethnic groups accounts for 45.84%, 40.48%, 6.50%, 0.83% of the total population respectively^11^. They have unique lifestyle and dietary habits due to special geographical environment and Muslim culture. Investigating on epidemic of MetS may reveal valuable information for making appropriate policies in preventive public health for them.

To date, most investigations of MetS in China have been conducted in mid-eastern China and Han ethnic group, and few studies cover the western China and multi-ethnic populations^12–14^. There have not been serious investigations to analyze the prevalence and risk factors of MS due to limited resources in public health and poor transportation in rural multi-ethnic region of Xinjiang. This study investigated the prevalence and the risk factors of MetS from a sample size of 15020 multi-ethnic adults in rural area of Xinjiang, making it one of the largest epidemiological surveys carried out in Xinjiang. The prevalence of MetS was estimated based on four widely used criteria in order to compare with the results in other studies.

## Results

### Prevalence of MetS and its components

**Figure 1:**
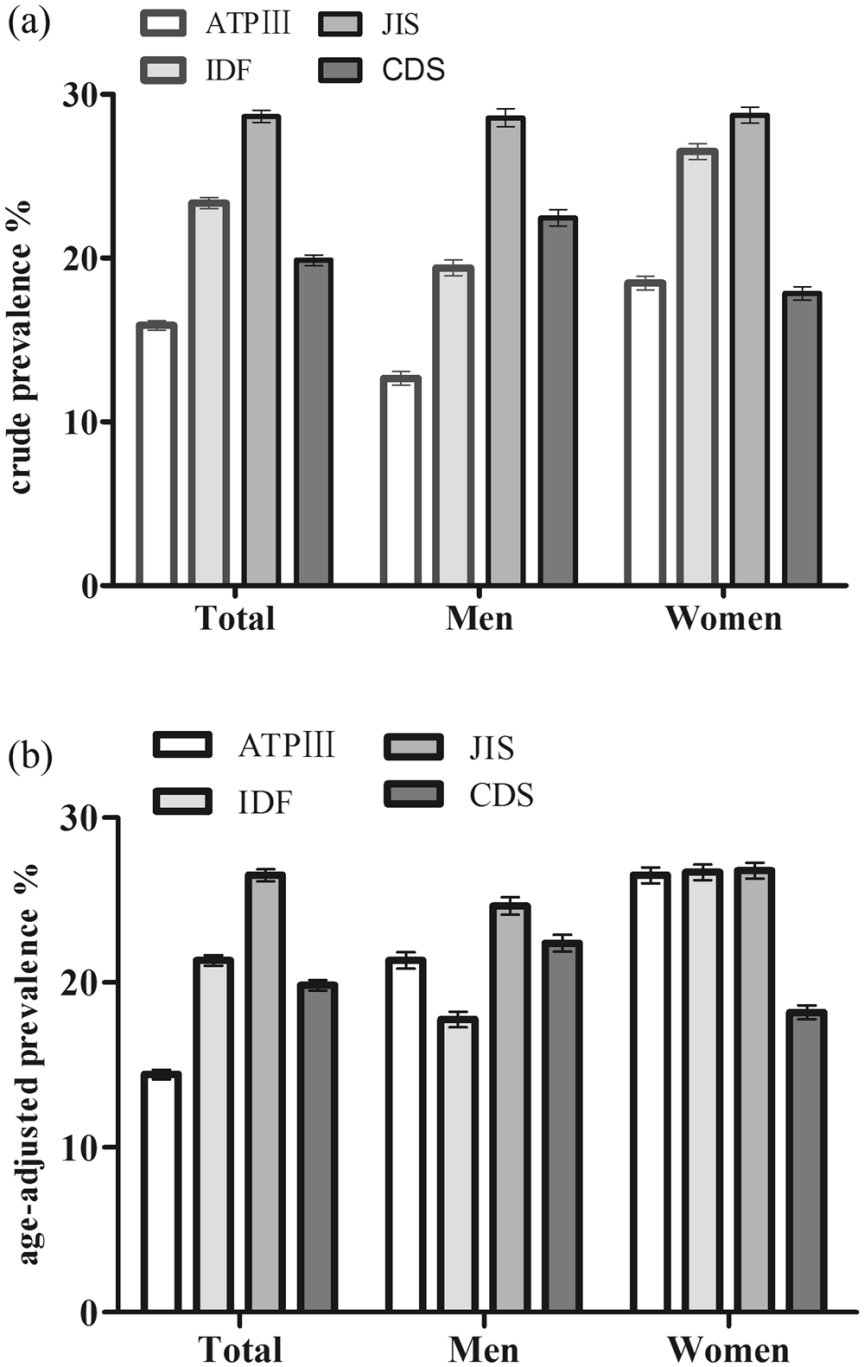
Crude and age-adjusted prevalence of MetS based on four diagnostic criteria in Xinjiang rural multi-ethnic adults. (**a**) Crude prevalence; (**b**) Age-adjusted prevalence; Data are presented as percentage (SE).

**Figure 2:**
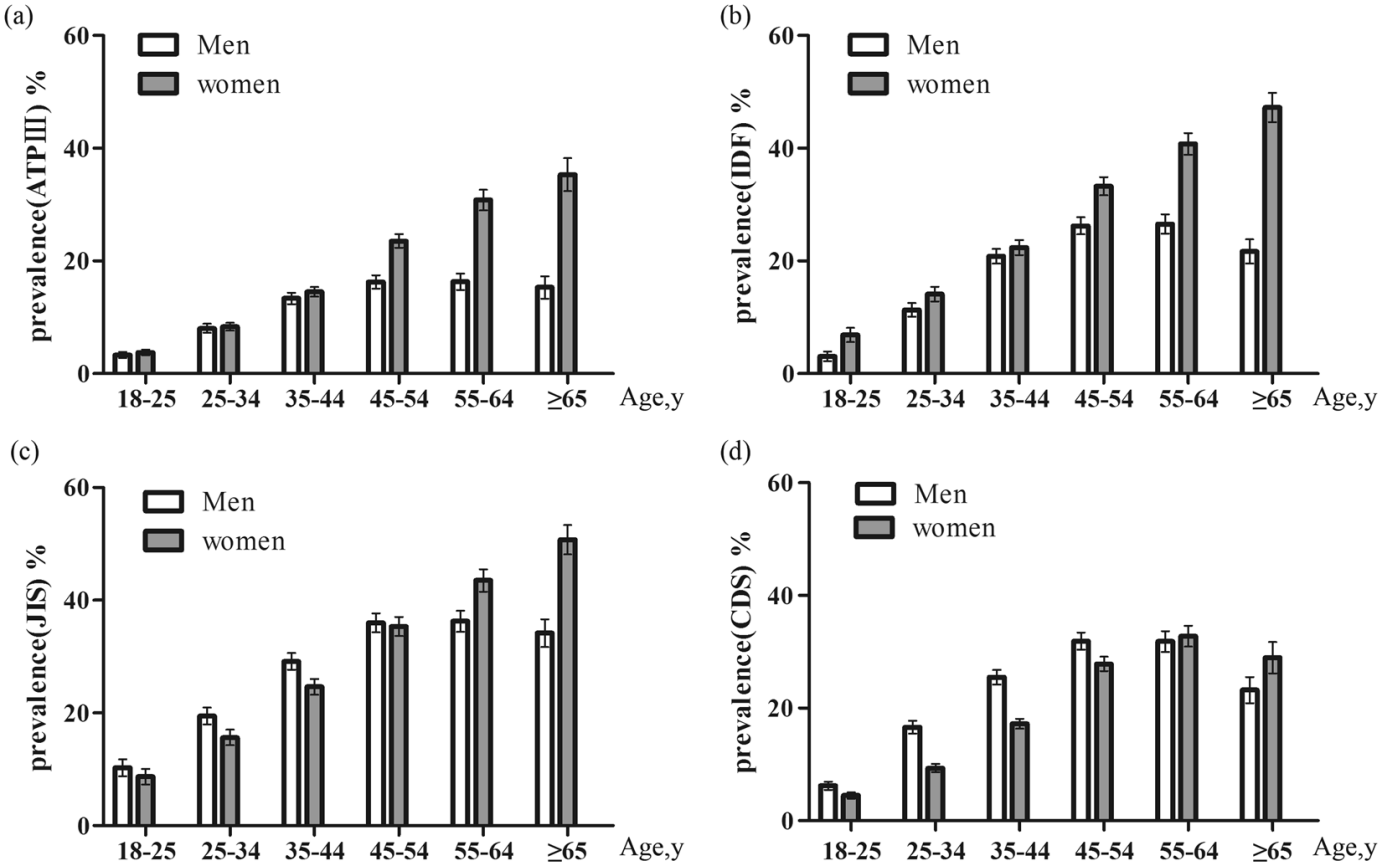
Age-specific prevalence of MetS based on four diagnostic criteria in Xinjiang rural multi-ethnic adults. (**a**) Prevalence by NCEP-ATPIII; (**b**) Prevalence by IDF; (**c**) Prevalence by JIS; (**d**) Prevalence by CDS; Data are presented as percentage (SE).

The overall crude prevalence of MetS was 15.90%, 23.36%, 28.66% and 19.89% according to the ATP III, IDF, JIS and CDS criterion, respectively. The age standardized prevalence was 14.43%, 21.33%, 26.50%, and 19.84%, respectively. The prevalence in women was higher than that in men by using the ATP III criterion (18.48% vs 12.66%, *P* < 0.001) and IDF definition (26.51% vs 19.41%, *P* < 0.001), but there was no significant difference based on the JIS criterion (28.57% vs 28.73%, *P* = 0.831). (Fig. 1). The prevalence of MetS increased with age in both men and women according to all of the criterion and peaked at 55–64 years in men and at ≥65 years in women (Fig. 2).

**Figure 3:**
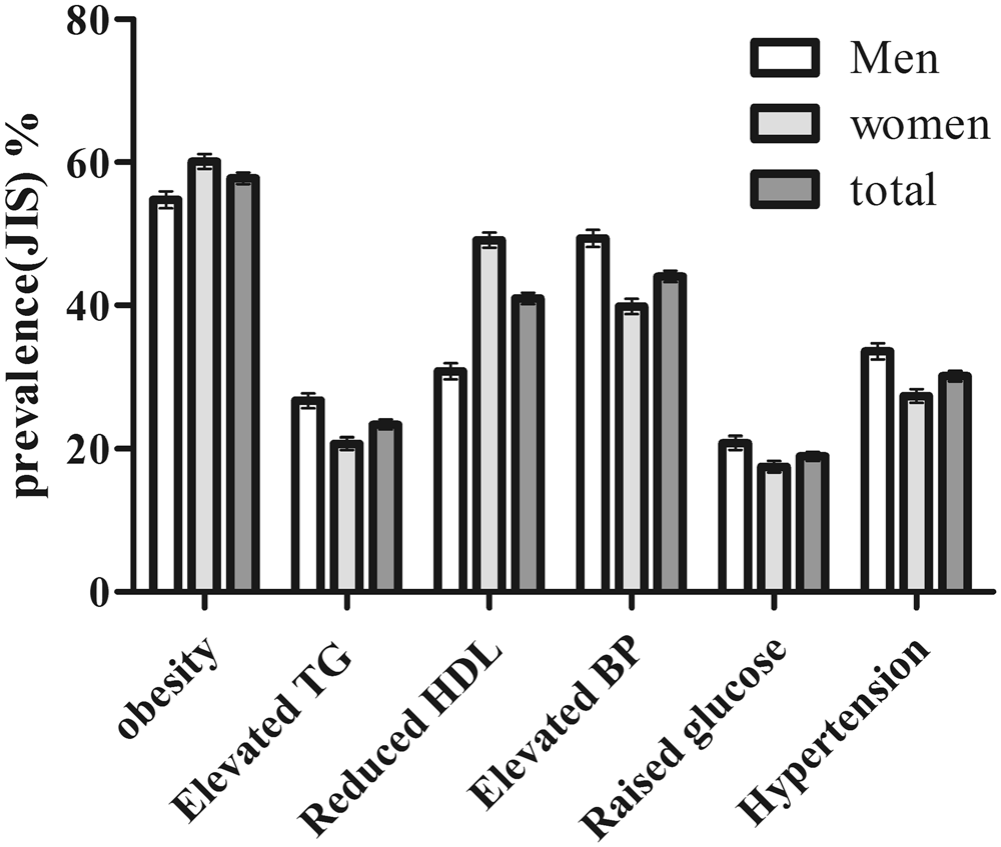
Prevalence of MetS components based on JIS criterion in Xinjiang rural multi-ethnic adults. Data are presented as percentage (SE).

According to the JIS criterion, The prevalence of components in MetS were 57.75% for abdominal obesity, 44.05% for elevated blood pressure, 40.98% for reduced HDL-cholesterol, 23.33% for elevated triglycerides, 18.95% for raised fasting plasma glucose. The most frequent individual component of MetS was abdominal obesity both in men (54.77%) and women (60.12%) followed by elevated blood pressure in men (49.35%) and reduced HDL-C in women (49.11%). There was a high prevalence of hypertension (total 30.09%, 33.56% in men and 27.32% in women) (Fig. 3).

### Characteristics of MetS cases and healthy individuals by JIS criterion

Compared with non-MetS cases, people in MetS cases were older and less educated, and the proportions of Kazakh and Han were higher than other ethnicities. In dietary factors, consumption of vegetable, fruits, fresh milk, and fresh meat were significantly different among MetS cases and non-MetS cases group (*P* < 0.05 for all). The rate of smoking and drinking in MetS cases group were higher than non-MetS cases group (Smoking, 33.52% vs 28.13%. Drinking, 24.58% vs 18.48%) (Table 1).

**Table 1:** Characteristics of MS cases and healthy individuals by JIS criterion (n/%).

Factors	MetS (n = 4305)	Non-MetS (n = 10715)	*P*
**Gender**			
Male	1903 (44.20)	4757 (44.40)	0.831
Female	2402 (55.80)	5958 (55.60)	
**Age (years)**			
18–24	150 (3.48)	1445 (13.49)	<0.001
25–34	468 (10.87)	2236 (20.87)	
35–44	968 (22.49)	2688 (25.09)	
45–54	1131 (26.27)	2044 (19.08)	
55–64	991 (23.02)	1481 (13.82)	
≥65	597 (13.87)	821 (7.66)	
**Ethnicity**			
Uyghur	807 (18.75)	2519 (23.51)	<0.001
Han	1114 (25.88)	2275 (21.23)	
Kazak	2131 (49.50)	4679 (43.67)	
Kyrgyz	253 (5.88)	1242 (11.59)	
**Marriage status**			
Yes	3650 (84.79)	9083 (84.77)	0.980
No	655 (15.21)	1632 (15.23)	
**Education**			
Illiteracy	900 (20.91)	1729 (16.14)	<0.001
Primary school	1963 (45.60)	4589 (42.83)	
≥Junior high school	1442 (33.50)	4397 (41.04)	
**Vegetables**			
no or <1 plate per week	820 (19.05)	1634 (15.25)	
1–3 plates per week	2271 (52.75)	5326 (49.71)	
≥4 plates per week	1214 (28.20)	3755(35.04)	<0.001
**fruits**			
no or <1 plate perweek	1046 (24.30)	2205 (20.58)	<0.001
1–3 plates per week	2031 (47.18)	5488(51.22)	
≥4 plates per week	1228 (28.52)	3022(28.20)	
**Fresh-milk**			
no or <0.5 L per week	3683 (85.55)	8942(83.45)	0.014
0.5–1.5 L per week	383 (8.90)	969(9.04)	
≥1.5 L per week	239 (5.56)	805 (7.51)	
**fresh meat**			
no or <1 kg per week	757 (17.58)	2332 (21.76)	<0.001
1–2 kg per week	1150(26.72)	3341 (31.18)	
≥2 kg per week	2398 (55.70)	5042 (47.06)	
**Salt**			
<500 g per month	1731 (40.20)	4250 (39.66)	0.667
≥500 g per month	2574 (59.80)	6465 (60.34)	
Drinking			
No	3247 (75.42)	8735 (81.52)	<0.001
Yes	1058 (24.58)	1980 (18.48)	
**Smoking**			
No	2862 (66.48)	7701 (71.87)	<0.001
Yes	1443 (33.52)	3014 (28.13)	

### Risk factors of MetS

According to the results of JIS criterion, we found that MetS was associated with age, education, consumption of vegetables, fruits, fresh milk, red meat, smoking, drinking by univariate analysis. The risk of MetS presents obvious trend of escalation with the increase of age. Compared with ≥18 years group, the value of odds ratio for MetS increased from 2.02 of 25- years group to 7.00 of ≥65 years group. The risk of MetS decreased 40% for the population with junior school degree or above compared with degree of illiteracy. 16.18% of participants intake ≥4 plates vegetables per week, 84.00% of participants didn’t drink fresh milk or less than 0.5 L per week, 49.52% of participants intake ≥2 kg fresh meat per week. Multiple-factors analysis presented that consumption of <1 plates vegetables and ≥2 kg red meat per week were associated with a higher risk of MetS (OR = 1.31, 95%CI: 1.12–1.54) and (OR = 1.64, 95%CI: 1.35–1.99), consumption of ≥1.5 L fresh milk per week were associated with a lower risk of MetS (OR = 0.64, 95%CI: 0.49–0.85) (Table 2).

**Table 2:** Risk factors for metabolic syndrome based on JIS criterion among Xinjiang rural multi-ethnic adults.

Factors	n (%)	OR(95%CI)^a^	OR(95%C)^b^	OR(95%C)^c^
**Gender**				
Male	6660 (44.34)	1.00	—	—
Female	8360 (55.66)	1.01 (0.94, 1.08)	—	—
**Age (years)**				
18–24	1595 (10.62)	1.00	—	—
25–34	2704 (18.00)	2.02 (1.66, 2.45)	—	—
35–44	3656 (24.34)	3.47 (2.89, 4.17)	—	—
45–54	3175 (21.14)	5.33 (4.44, 6.40)	—	—
55–64	2472 (16.46)	6.45 (5.35, 7.77)	—	—
≥65	1418 (9.44)	7.00 (5.74, 8.54)	—	
**Ethnicity**				
Uyghur	3326 (22.14)	1.00	—	—
Han	3389 (22.56)	1.53 (1.37, 1.70)	—	—
Kazak	6810 (45.34)	1.42 (1.29, 1.56)	—	—
Kyrgyz	1495 (9.95)	0.64 (0.54, 0.74)	—	—
**Marriage status**				
Yes	12733 (84.77)	1.00	—	—
No	2287 (15.23)	1.00 (0.91, 1.10)	—	—
Education				
Illiteracy	2629 (17.50)	1.00	—	—
Primary school	6552 (43.62)	0.82 (0.75, 0.90)	—	—
≥Junior high school	4189 (27.89)	0.63 (0.57, 0.70)	—	
**Vegetables**				
≥4 plates per week	5012 (33.37)	1.00	1.00	1.00
1–3 plates per week	7578 (50.45)	1.32 (1.16, 1.50)	1.36 (1.19, 1.56)	1.38 (1.19, 1.61)
no or <1 plate per week	2430 (16.18)	1.55 (1.31, 1.84)	1.61 (1.34, 1.92)	1.64 (1.35, 1.99)
**fruits**				
no or <1 plate per week	3255 (21.67)	1.00	1.00	—
1–3 plates per week	7515 (50.03)	0.78 (0.71, 0.86)	0.93 (0.84, 1.03)	—
≥4 plates per week	4251 (28.30)	0.86 (0.77, 0.95)	0.93 (0.84, 1.03)	—
Fresh milk				
no or <0.5 L per week	12617 (84.00)	1.00	1.00	1.00
0.5–1.5 L per week	1352 (9.00)	0.96 (0.80, 1.15)	0.96 (0.78, 1.15)	0.81 (0.64, 1.04)
≥1.5 L per week	1050 (6.99)	0.72 (0.58, 0.90)	0.68 (0.54, 0.85)	0.64 (0.49, 0.85)
**fresh meat**				
no or <1 kg per week	3090 (20.57)	1.00	1.00	1.00
1–2 kg per week	4491 (29.90)	1.06 (0.94, 1.19)	1.06 (0.94, 1.20)	1.14 (0.98, 1.32)
≥2 kg per week	7438 (49.52)	1.47 (1.32, 1.63)	1.36 (1.22, 1.52)	1.31 (1.12, 1.54)
**Salt**				
<500 g per month	5979 (39.81)	1.00	—	—
≥500 g per month	9041 (60.19)	0.98 (0.88, 1.08)	—	—
**Drinking**				
No	11982 (79.77)	1.00	1.00	1.00
Yes	3038 (20.23)	1.44 (1.32, 1.56)	1.23 (1.12, 1.34)	0.98 (0.76, 1.27)
**Smoking**				
No	10563 (70.33)	1.00	1.00	1.00
Yes	4457 (29.67)	1.29 (1.19, 1.39)	1.13 (1.05, 1.22)	1.08 (0.92, 1.26)

Note: ^a^Non-adjusted, ^b^Adjusted for age, education, minority. ^*c*^Adjusted for age, education, minority and significant factors in model *b*.

## Discussion

Although there have been many epidemiology investigations on MetS, few studies have focused on rural multi-ethnic populations. This survey was conducted in typical multi-ethnic rural areas in Xinjiang where approximately 95% of the populations were the Uyghur, the Han, the Kazakh and the Kyrgyz. They have their own special genetic characteristics and lifestyle which are quite different from either the Hans in inland provinces of China or American/European populations^15,16^. According to three most widely used criteria, the age-standardized prevalence of MetS were 14.43% (ATPIII), 21.33% (IDF), and 26.50% (JIS), respectively. The prevalence rate was higher than that of the national level in China (18.2%, IDF)^6^, Japan(13.3%, IDF)^17^,and rural low-income region of Africa (22.1%, JIS)^18^, and lower than that in Spain (33.2%, JIS)^19^ and the USA (34.3%, JIS)^20^. The high prevalence of MetS in Xinjiang rural multi-ethnic residents may be associated with high prevalence of hypertension and dyslipidemia^21,22^. This study found that the prevalence of elevated blood pressure and reduced HDL-cholesterol were 44.05% and 40.98%, respectively. It was higher than the average level of Chinese general residents^23,24^.

Many studies have found that the prevalence of MetS increases with age without an inflection point. Although the prevalence of MetS in Xinjiang rural multi-ethnic residents also increased with age, it appeared to decline after 65 years old. The possible reason for this was that a large number of patients died before 65 due to the high mortality related with cardiovascular diseases^25,26^. Our study confirmed that the risk of MetS was not reduced after 65 years old compared with previous age group. Previous studies have shown that MetS in Chinese women is more widespread than that in men^6,27^. Our study also found that the prevalence in women was higher than that in men by using all of three criteria. We found an interesting trend in which the discrepancy between women and men decreased from ATP III to IDF and IDF to JIS.

We found that the dietary factors were strongly associated with the prevalence of MetS in Xinjiang rural multi-ethnic populations. Consumption less vegetable and much fresh meat were associated with a higher risk of MetS, but consumption much fresh milk was associated with a lower risk of MetS. Martini found that the prevalence of MetS in individuals who reported not consuming regularly vegetables was approximately two times higher than their peers who reported adequate intake^28^. A study from Korean also found that having greater total vegetables was associated with a lower risk of MetS (OR = 0.47, 95%CI: 0.29–0.75)^29^. The underlying mechanism may be that vegetables intake was associated with blood pressure and glucose levels. A review reported vegetable intake was associated with a reduction in diastolic blood pressure^30^. A perspective study revealed that vegetarian dietary patterns have been associated with a lower risk for developing type 2 diabetes, hypertension^31^.

Xinjiang rural residents maintain a high-energy diet due to cold climate and heavy labor. They consume greater amounts of meat, pasta, dairy foods (cheese and milk tea), but less vegetables compared with the residents in other regions of China^32^. Our results indicated that only 16.18% of participants intake ≥4 plates vegetables per week. The less consumption of vegetables may lead to the high prevalence of MetS in them. Less consumption of vegetables was related to geography and climate conditions in Xinjiang, in which six months of the year are as cold as in winter. It is difficult to plant and store fresh vegetables, and the vegetables from out of town are too expensive for them to buy. It is more difficult for rural residents to get fresh vegetables due to low income and inconvenient transportation. Although vegetables are not easy to get for Xinjiang rural residents, fresh milk is already available due to the most of survey areas were pastoral areas. This research showed that the risk of having MetS was decreased by 32% if a person drink ≥1.5 L fresh milk weekly compared to those who do not drink fresh milk. While most of rural residents eat dairy foods, such as cheese, milk tea, koumiss, few of them drink fresh milk directly. Our study revealed that 84% of participants didn’t drink fresh milk or less than 0.5 L per week. Therefore promoting consumption of fresh milk may be a practical and feasible measure to prevent MetS in Xinjiang rural multi-ethnic residents.

The fresh meats that Xinjiang residents consumed are mainly red meat including beef and mutton. Red meat contains high levels of saturated fat, cholesterol, iron contents. Consumption of saturated fat was associated with components of MetS, such as elevated blood pressure and dyslipemia^33,34^. Red meat was also related to the deposition of iron while iron overload was positively associated with insulin resistance, which was one of underlying mechanisms for MetS^35^. In addition, red meat intake was associated with a chronic state of inflammation that appears to be a central mechanism underlying the pathophysiology of MetS^36^. Previous study indicated that there was a direct relationship between red meat intake and elevated risk of metabolic syndrome^37^. This study also confirmed that too much red meat intake might increase risk of MS. Red meat consumption among Xinjiang rural residents is above the national average due to the more developed animal husbandry^38^. It is one of possible reasons that the prevalence of MetS in Xinjiang rural residents is higher than that of the national level.

The strengths of this study include a large sample size in multi-ethnic rural area of Xinjiang, and a comprehensive data were collected to analyze the prevalence and risk factors of MetS. However, limitations should also be taken into account. First, the cross-sectional study design precluded casual conclusions and further longitudinal researches were needed to determine the causal relationship. Second, some of younger men moved out of area to work during enrollment and they cannot be included in this study. It could impact the analysis of younger people. Finally, the energy intake and fat percentage may affect the associations between diet and MetS, but they could not be calculated by food frequency method in our study. These factors should be analyzed as covariates in the future researches.

In conclusion, the prevalence of MetS in Xinjiang rural adults was higher than that of national average level of China and falls in between the Euro-American and Asia levels. Less consumption of vegetables and much intake of red meat were the main risk factors of MetS. While increasing intake of fresh milk would be an optional measure to reduce risk of MetS in them.

## Materials and Methods

### Settings and Participants

This study was conducted from 2009 to 2010 in Xinjiang. Multi-stage (prefecture-county-township-village) stratified cluster random sampling was employed to choose participants. First, four representative prefectures (Kashi, Shihezi,Yili and Kizilsu) were identified based on the population distribution of the Uyghur, Hans, Kazakhs, and Kyrgyz. Secondly one county was randomly selected in each prefecture and one township from each county. Finally, a cluster sampling method was used to select the corresponding villages in each township. The residents of selected villages were investigated. The selection criteria were as follows: (1) People who are living in the village for at least 6 months. (2) People with clear consciousness and without mental diseases. (3) Women who are not pregnant. (4) People who are able to perform anthropometric measurements. (5) People who are willing to cooperate with the investigation. A total of 15,020 (6660 men and 8360 women) participants met the selection criteria and were asked to complete questionnaires, anthropometric measurements and blood tests. The overall response rate was 89.6% (91.5% for Uyghurs, 87.1% for Kazakhs, 90.3% for Hans and 93.5% for Kyrgyz, respectively).

### Data collection

Data were collected by trained graduate students using a standard questionnaire in a face-to-face interview during field investigation. The questionnaires included the demographic characteristics, diet, lifestyle risk-factors, family income and history of chronic diseases. A validated quantitative food frequency questionnaire was designed to collect information of the dietary intakes in the past week^39^. The questionnaire included information about the consumption of 12 food groups. These food groups were as follows: vegetables, fruits, fresh milk, red-meat, dairy product, pasta, eggs, sea-foods, viscera, bean products, fried foods, salt. The unit of the vegetables and fruits intake per week was measured by plates (diameter 16 cm). The unit of red meat was measured by kilogram (kg). Fresh milk was measured by liter (L). Smokers were defined as participants who had smoked 100 cigarettes and smoke now^40^. Drinkers were defined as participants who intake alcohol beverages (liquor, beer and grape wine) at least 2 times per month and drink now^10^. Blood pressure and waist circumference were measured by trained investigators following a standard protocol^41^. The blood pressure was measured using a mercury sphygmomanometer. The measurements were collected in triplicate after a 5-minute seated rest and were averaged as the blood pressure values of the individual. The waist circumference was defined as the midpoint between the lower rib and upper margin of the iliac crest at minimal respiration, as measured by a non-elastic ruler tape with an insertion buckle at one end to the nearest 0.1 cm.

### Blood plasma glucose and lipid measurements

A 5-ml fasting blood sample was collected from each participant. The sample was centrifuged at 3000 rpm for 30 min, and plasma was stored at −80 °C. The serum glucose, HDL-cholesterol and triglycerides were tested by a modified hexokinase enzymatic method using an Olympus AV2700 Biochemical Automatic Analyzer (Olympus, Japan) in the Biochemistry Laboratory, the First University-Affiliated Hospital of Shihezi University School of Medicine. Quality control was strictly followed by the procedure of blood collection, storage, and measuring processes.

### Definitions

Three world-wide definitions and one Chinese criterion were used to diagnose MetS. These definitions were from the Joint Interim Statement of multi-organizations in 2009 (JIS)^42^, the International Diabetes Federation world-wide definition in 2005 (IDF)^4^, the Third Report of the National Cholesterol Education Program Expert Panel on Detection, Evaluation, and Treatment of High Blood Cholesterol in Adults in 2001 (ATPIII)^43^, and Chinese criterion was suggested by the Chinese Diabetes Society in 2004 (CDS)^44^. JIS criterion was the primary definition to examine risk factors of MetS. The detailed information on the four criteria was listed in Supplementary Table S1.

### Statistical Analysis

Categorical variables were presented as numbers and percentages. Differences between groups were tested using Chi-square test. MetS prevalence was calculated for the total adult population and the age groups 18–24, 25–34, 35–44, 45–54, 55–64, ≥65. The method of direct standardization was used to calculate the age-standardized prevalence of MetS. The calculation was weighted based on Chinese population data from 2010 census^45^. All statistical tests were two-sided. *P* value < 0.05 was considered statistically significant. Univariate logistic regression was used to find significant variables associated with MetS according to JIS criterion. Multiple factor analysis was performed to identify the association between MetS and dietary factors, smoking and drinking. We established a database using EpiData software (EpiData Association, Odense, Denmark, http://www.epidata.dk/). The data were analyzed using SPSS (Statistical Program for Social Sciences, version 13.0, 2004) and Empower Stats.

### Ethical approval

This study has been approved by The Institutional Ethics Review Board (IERB) at the First Affiliated Hospital of Shihezi University School of Medicine (IERB No. SHZ2010LL01). All procedures performed in studies involving human participants and experiments were in accordance with the approved guidelines and regulations. And that the written informed consent was obtained from each participant.

### Data availability

The datasets generated during the current study are available from the corresponding author on reasonable request.

## Electronic supplementary material

Supplementary Table
