# Supplementary material for: Prevalence of Metabolic Syndrome and its Associated Factors among Multi-ethnic Adults in Rural Areas in Xinjiang, China
Source: Sci Rep. 2017 Dec 15;7:17643. doi: 10.1038/s41598-017-17870-5 (PMC5732195; doi:10.1038/s41598-017-17870-5)
Supplement: Supplementary file 1 — Supplementary Table [file 41598_2017_17870_MOESM1_ESM.doc]

**Prevalence of Metabolic Syndrome and Its Associated Factors among** **Multi-ethnic Adults in Rural Areas in Xinjiang, China**

**Heng Guo, ^1^ Xiang Gao,^3^ Rulin Ma,^2^ Jiaming Liu,^2^ Yusong Ding, ^2^ Mei Zhang, ^2^** **Jingyu Zhang, ^2^ Lati Mu, ^2^ Jia He, ^2^ Yizhong Yan, ^2^ Jiaolong Ma, ^2^ Shuxia Guo*, ^2^ Sheng Wei*^1^**

Author affiliations:

1 Department of Epidemiology and Biostatistics, School of Public Health, Tongji Medical College, Huazhong University of Science and Technology, Wuhan, Hubei 430030, China.

2 Department of Public Health, Shihezi University School of Medicine, Shihezi, Xinjiang 832000, China.

3 Department of Nutritional Sciences, The Pennsylvania State University 109 Chandlee Lab, University Park, PA 16801, USA.

Corresponding author A: Sheng Wei,

Sheng Wei, MD, PhD. Department of Epidemiology and Biostatistics, School of Public Health, Tongji Medical College, Huazhong University of Science and Technology, 13 Hangkong Road, Wuhan, Hubei, 430030, P.R. China.

Tel: 86-27-83692031; Fax: 86-27-83692031;

E-mail: ws2008cn@gmail.com

Corresponding author B: Shuxia Guo

Tel: 86-993-2057153; Fax: 86-993-2057153;

E-mail: pge888@sina.com

**Supplementary Table S1** Definitions of metabolic syndrome.

| **Risk factors** | **ATP Ⅲ criterion(2001)** | **IDF criterion(2005)** | **JIS criterion(2009)** | **CDS criterion(2004)** |
| --- | --- | --- | --- | --- |
| Abdominal obesity  (Chinese) | Waist circumference:  men≥102 cm  women≥88 cm | Waist circumference:  men≥90 cm  women≥80 cm | Waist circumference:  men≥85 cm  women≥80 cm | BMI≥25 (Kg/m^2^) |
| Raised triglycerides | ≥150 mg/dL (1.7 mmol/L) | ≥1.7 mmol/L(150 mg/dl)  or treatment for this lipid abnormality | ≥150 mg/dl (1.7 mmol/L) or treatment for this lipid abnormality | ≥150 mg/dl (1.7 mmol/L) |
| Reduced HDL-cholesterol | <40 mg/dL (1.03 mmol/L) in men; <50 mg/dL (1.29 mmol/L) in women | < 1.03 mmol/l (40 mg/dl) in men; <1.29 mmol/L (50 mg/dl) in women; or treatment for this lipid abnormality | <40 mg/dl (1.0 mmol/L) in men; <50 mg/dl(1.29 mmol/L) in women or treatment for this lipid abnormality | <35mg/dl (0.9 mmol/L) in men; <39 mg/dl (1.0mmol/L) in women |
| Elevated blood pressure | Systolic≥ 130 mm Hg or/and  Diastolic≥85 mm Hg | Systolic: ≥ 130 mmHg or/and  Diastolic: ≥ 85 mmHg  or treatment of previously diagnosed hypertension. | Systolic≥ 130 mm Hg or/and Diastolic≥85 mm Hg or treatment of previously diagnosed hypertension. | Systolic≥ 140 mm Hg or/and Diastolic≥90 mm Hg or treatment of previously |
| Raised fasting plasma glucose | ≥ 110 mg/dl | ≥ 5.6 mmol/L (100 mg/dl)  or previously diagnosed type 2 diabetes | ≥ 100 mg/dl | ≥ 6.1mmol/L (110 mg/dl)  or previously diagnosed type 2 diabetes |

**Note:** Metabolic syndrome was diagnosed: the ATP Ⅲ criterion: a person had at least three of the five factors; the IDF criterion: a person had abdominal obesity plus 2 or more other risk factors; the JIS criterion: a person had at least three of the five factors; the CDS criterion: a person had at least three of the five factors.
